# Differential Trypanosome Surface Coat Regulation by a CCCH Protein That Co-Associates with *procyclin* mRNA *cis*-Elements

**DOI:** 10.1371/journal.ppat.1000317

**Published:** 2009-02-27

**Authors:** Pegine Walrad, Athina Paterou, Alvaro Acosta-Serrano, Keith R. Matthews

**Affiliations:** 1 Centre for Immunology, Infection and Evolution, Institute of Immunology and Infection Research, School of Biological Sciences, Ashworth Laboratories, University of Edinburgh, Edinburgh, United Kingdom; 2 Wellcome Centre for Molecular Parasitology, University of Glasgow, Glasgow, United Kingdom; Yale University, United States of America

## Abstract

The genome of *Trypanosoma brucei* is unusual in being regulated almost entirely at the post-transcriptional level. In terms of regulation, the best-studied genes are *procyclins*, which encode a family of major surface GPI-anchored glycoproteins (EP1, EP2, EP3, GPEET) that show differential expression in the parasite's tsetse-fly vector. Although *procyclin* mRNA *cis*-regulatory sequences have provided the paradigm for post-transcriptional control in kinetoplastid parasites, *trans*-acting regulators of *procyclin* mRNAs are unidentified, despite intensive effort over 15 years. Here we identify the developmental regulator, *Tb*ZFP3, a CCCH-class predicted RNA binding protein, as an isoform-specific regulator of Procyclin surface coat expression in trypanosomes. We demonstrate (i) that endogenous *Tb*ZFP3 shows sequence-specific co-precipitation of *EP1* and *GPEET*, but not *EP2* and *EP3*, *procyclin* mRNA isoforms, (ii) that ectopic overexpression of *Tb*ZFP3 does not perturb the mRNA abundance of *procyclin* transcripts, but rather that (iii) their protein expression is regulated in an isoform-specific manner, as evidenced by mass spectrometric analysis of the Procyclin expression signature in the transgenic cell lines. The *Tb*ZFP3 mRNA–protein complex (*Tb*ZFP3mRNP) is identified as a *trans*-regulator of differential surface protein expression in trypanosomes. Moreover, its sequence-specific interactions with *procyclin* mRNAs are compatible with long-established predictions for Procyclin regulation. Combined with the known association of *Tb*ZFP3 with the translational apparatus, this study provides a long-sought missing link between surface protein *cis*-regulatory signals and the gene expression machinery in trypanosomes.

## Author Summary

Trypanosomes, the tropical parasites that cause African sleeping sickness, show a number of biological peculiarities that distinguish them from other eukaryotes. One is the unusual way in which they regulate gene expression. Unlike most eukaryotes, trypanosomes do not regulate gene expression by controlling the rate of messenger RNA synthesis, but, instead, control the stability of messenger mRNAs (and, hence, their abundance) and also their rate of translation into protein. The best-studied model for this “post-transcriptional” gene expression control in trypanosomes is the *procyclin* mRNAs, which encode the major surface proteins of the parasite in the tsetse fly. In this study we demonstrate that a small kinetoplastid-specific protein (*Tb*ZFP3) co-associates with the mRNAs for some *procyclin* isoforms (*EP1*, *GPEET procyclin*) but not others (*EP2*, *EP3 procyclin*). Furthermore, we show that this is dependent upon sequences in the *EP1 procyclin* 3′untranslated region known to govern its mRNA turnover and protein synthesis. Finally, we demonstrate that limited over-expression of *Tb*ZFP3 causes a change in the surface protein expression profile on cultured parasites from GPEET to EP1 Procyclin. Our data identify *Tb*ZFP3 as an important post-transcriptional regulator of Procyclin expression, the first such protein factor identified.

## Introduction

The pathway of mRNA control in eukaryotes involves regulatory steps at multiple stages. This is reflected by the large investment of eukaryotic genomes in RNA binding proteins, with hundreds of genes in yeasts and mammals being devoted to functions requiring RNA interaction. As the diverse roles of these proteins, and their interactions with specific subsets of mRNAs, are investigated, it is becoming clear that post-transcriptional control represents a regulatory network of a complexity and importance likely greater than transcriptional control [1].

Perhaps the most extreme example of a group of organisms with emphasis on post-transcriptional control is the kinetoplastid parasites. These organisms are of medical, veterinary and economic importance because they are responsible for an enormous burden of disease within the tropics, including a variety of cutaneous and visceral diseases (caused by *Leishmania* spp.), Chagas disease (caused by *Trypanosoma cruzi*) and African sleeping sickness (caused by *Trypanosoma brucei*). Here, an absence of detectable RNA II polymerase promoters for protein coding genes and the general organisation of transcription units into polycistronic arrays necessitates almost complete reliance on post-transcriptional control for regulated gene expression [2]. Supporting this, the genome of these parasites reveals a complexity and composition of encoded RNA binding proteins exceeding, and distinct from, that found in the crown group of eukaryotic organisms [3],[4].

Gene regulation is particularly important in kinetoplastid parasites because their life cycle is complex, involving passage through a mammalian host and within distinct compartments of an arthropod vector [5]. One of the best-characterised life-cycle differentiation events involves exchange of the major surface antigens as African trypanosomes passage from mammalian blood to the midgut of their haematophagous vector, the tsetse fly [6]. In the bloodstream trypanosomes stay ahead of the immune response by expressing, sequentially and hierarchically, thousands of different antigenic surface coats comprised of variant surface glycoprotein (VSG) [7]. However, upon differentiation in the tsetse, the VSG coat is replaced by a family of glycophosphatidyl inositol (GPI)-anchored proteins known as Procyclins. There are two types of Procyclin proteins, which mainly differ by the type of amino acid repeats they contain at their C-termini. One set of proteins, the EP isoforms (encoded by the *EP1-1*, *EP1-2*, *EP2* and *EP3* genes) contain 22–30 [E-P] internal repeat peptides whereas GPEET Procyclins (encoded by one copy of *GPEET*) contain 6 [G-P-E-E-T] repeats, which can be phosphorylated at the Thr residues. In the tsetse, Procyclins follow a programmed expression and their C-terminal repeat peptides, together with their complex GPI anchors, may provide protection for the parasite from the action of tsetse gut hydrolases [8]–[11].

Sequence-dependent signals in the 3′ untranslated region (3′ UTR) of each *procyclin* mRNA govern their expression and have been the subject of intense investigation, providing the paradigm for gene expression control in kinetoplastid parasites [2]. Although the 3′UTRs of *EP1*, *2* and *3* and *GPEET procyclin* mRNAs are highly similar, the genes are differentially regulated in distinct phases of tsetse infection or *in vitro*. For example, the *GPEET procyclin* 3′UTR contains an element, absent in the closely related *EP1* 3′UTR, that differentially regulates its expression in response to glycerol and the activity of mitochondrial enzyme activities [12],[13]. However, whilst the *cis*-acting control sequences for *procyclin* mRNAs are very well characterised [14]–[17], protein factors that recognise these regulatory domains have remained unidentified, despite considerable effort. Here we establish the specific association and regulation of *procyclin* mRNA isoforms by a kinetoplastid-specific protein factor that associates with polyribosomes, providing the first example in these organisms of surface protein regulation by an mRNA-associated regulatory factor.

## Results

### *Tb*ZFP3 immunoprecipitation differentially selects *procyclin* isoform mRNAs

**Figure 1:**
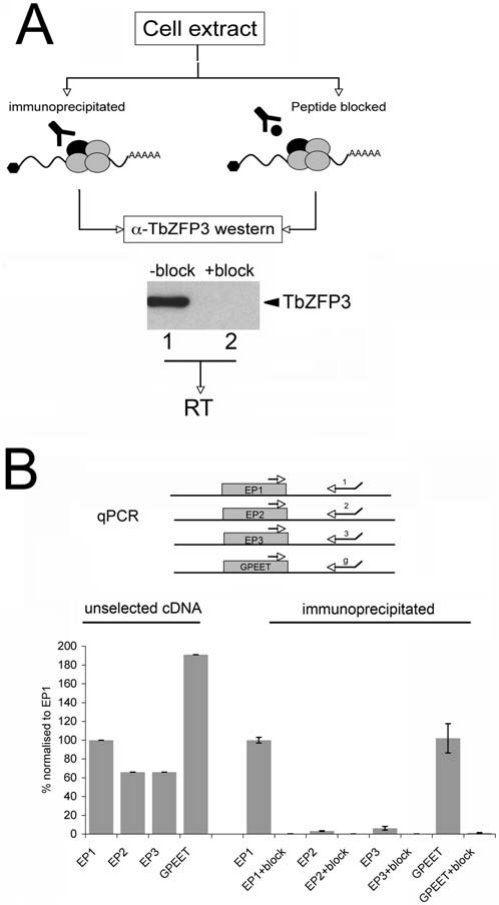
Co-immunoprecipitation of *EP1* and *GPEET procyclin* mRNA, but not *EP2* and *EP3* mRNA, by *Tb*ZFP3. (A) Schematic representation of *Tb*ZFP3 immunoprecipitation. Cell extracts were subject to a clearing spin and immunoprecipitated in the presence or absence of the peptide immunogen against which the *Tb*ZFP3 antibody was raised (“Peptide blocked”). Western blotting with anti-serum to *Tb*ZFP3 was used to detect selection in the absence (Lane 1) or presence (Lane 2) of blocking peptide. (B) Three replicate immunoprecipitations from three distinct trypanosome procyclic lines were carried out, with the selected material being subject to reverse transcription and amplification using primers specific for *EP1*, *EP2*, *EP3*, or *GPEET* mRNAs. Values are normalised in unselected and selected material to the level of *EP1* mRNA. Error bars = SD.

Previous immunoprecipitation experiments using an antibody specific for a small CCCH-protein implicated in developmental control, *Tb*ZFP3 (Tb927.3.720), demonstrated co-precipitation of mRNA for the procyclic-form specific surface proteins, Procyclins [18]. Since different Procyclin isoforms exhibit distinct profiles of mRNA and protein expression in the tsetse fly [8],[11],[19] we investigated whether each isoform mRNA was co-precipitated with equivalent efficiency by *Tb*ZFP3. Figure 1A shows a typical experiment where immunoprecipitation from cell extracts resulted in a selection for *Tb*ZFP3 (lane 1), this being blocked in the presence of the peptide immunogen used to raise the *Tb*ZFP3-specific antibody (lane 2). The resulting co-selected mRNAs were then reverse transcribed and subjected to quantitative real-time (qRT) PCR using primers specific for each *procyclin* transcript isoform [11] (Figure 1B). In parallel reactions, total RNA from the starting cultures was also analysed with each primer set, allowing us to compare the relative level of each *procyclin* isoform mRNA in unselected and *Tb*ZFP3-immunoprecipitated material. In total mRNA of the cell extracts, both *EP2* and *EP3* were present at 66% of *EP1* levels (*EP1* is normalised to 100% in Figure 1B), approximating to their observed relative abundance in culture and in the tsetse midgut [11]. As expected in this parasite strain [12],[20],[21], *GPEET* mRNA was also abundant (191% with respect to *EP1*) in the unselected material. In contrast to unselected cDNA, *Tb*ZFP3-immunoprecipitated material showed a strikingly differential abundance of the isoforms, such that *EP1* and *GPEET* were the dominant selected transcripts, with *EP2* and *EP3* selected at much lower level (3.3% and 6.2% of immunoprecipitated *EP1*, respectively). Importantly, use of the peptide block prevented the immunoprecipitation of each *procyclin* mRNA isoform, demonstrating specificity of the selection. Supporting this qRT-PCR data, non-selective amplification and cloning of *procyclin* cDNAs derived from *Tb*ZFP3-immunoprecipitated material isolated 21/27 (78%) *EP1* sequences and 6/27 (22%) *GPEET* sequences, with no clones containing *EP2* or *EP3* derived sequences. We conclude that although *EP1*, *EP2*, *EP3* and *GPEET procyclin* mRNAs are each abundant in the unselected mRNA pool, *Tb*ZFP3 is preferentially associated with *EP1* mRNA, but also *GPEET procyclin* mRNAs.

### Procyclin mRNA association requires integrity of the CCCH domain in *Tb*ZFP3

**Figure 2:**
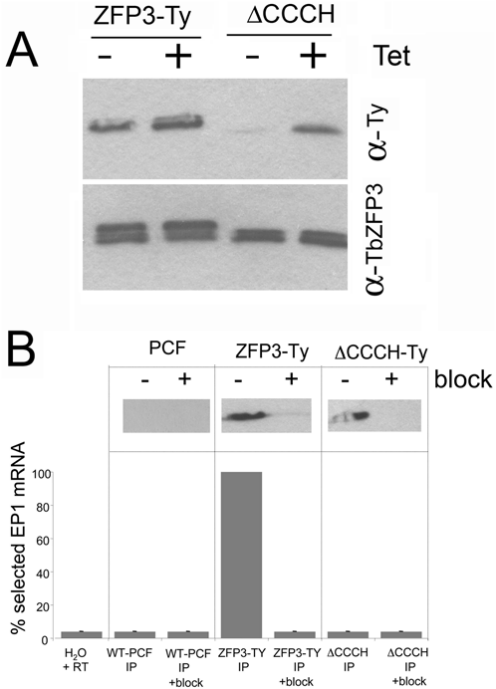
The CCCH domain of *Tb*ZFP3 is necessary for *EP1 procyclin* mRNA co-association. (A) Western blot of the expression of the Ty-tagged ΔCCCH mutant of *Tb*ZFP3 and the Ty-tagged wild type *Tb*ZFP3 expressed in transgenic parasites. The ectopically expressed proteins were detected by incorporation of the Ty-1 epitope tag in to the transgenic protein, allowing their detection and immunoprecipitation. The upper panel shows cell extracts reacted with anti-Ty1 epitope tag antibody BB2, the lower panel shows the same cell extracts reacted with anti-*Tb*ZFP3 antibody. This detects the endogenous *Tb*ZFP3 as well as the ectopically expressed, Ty-tagged copy. Note that the *Tb*ZFP3-Ty migrates more slowly than the endogenous protein due to incorporation of the 10 amino acid epitope tag, whereas the ΔCCCH mutant comigrates with the endogenous protein due to deletion of the CCCH domain. (B) RNA immunoprecipitation of *EP1 procyclin* mRNA using the BB2 antibody to select *Tb*ZFP3-Ty or ΔCCCH ZFP3 ectopic protein from cell extracts. The relative selection of *EP1* mRNA is normalised to selection with *Tb*ZFP3-Ty in each case. The relative immunoprecipitation of each ectopic protein in the presence or absence of a Ty1-epitope specific peptide block is shown at the top of each sample to demonstrate the efficiency and specificity of immunoprecipitation. This represents a Western blot of the immunoprecipitated material reacted with the BB2 antibody, specific for the Ty-1 epitope tag incorporated into the ectopically expressed *Tb*ZFP3-Ty or *Tb*ZFP3 ΔCCCH.

To determine whether the co-immunoprecipitation of *procyclin* mRNAs with *Tb*ZFP3 was dependent on its predicted RNA-binding domain we examined a cell line expressing a mutant form of *Tb*ZFP3 lacking the CCCH zinc finger domain (*Tb*ZFP3 ΔCCCH; [18]). This mutant incorporated a C-terminal Ty1-epitope tag to allow it to be specifically immunoprecipitated in the context of endogenous *Tb*ZFP3 using the BB2 antibody which detects the Ty1 epitope [22]. As a control, wild type *Tb*ZFP3 was also expressed with a C-terminal Ty1 tag with the relative expression of each ectopically expressed protein being examined by Western blotting using either the antibody against *Tb*ZFP3 (this detecting endogenous and ectopically expressed *Tb*ZFP3) or BB2 (detecting only the ectopically expressed protein). Figure 2A shows the relative expression of each ectopically expressed protein in each cell line, confirming their approximately equivalent abundance. Thereafter, cell extracts from each line were used in immunoprecipitation experiments to select the ectopic *Tb*ZFP3 using the BB2 antibody, and the co-selection of *procyclin EP1* mRNA assayed. This demonstrated selection of *EP1* mRNA with *Tb*ZFP3-Ty, as expected, whereas deletion of the CCCH domain prevented co-precipitation of *EP1* mRNA (Figure 2B). Thus, the integrity of the predicted RNA binding domain in *Tb*ZFP3 is necessary for co-immunoprecipitation of *EP1* mRNA.

### Sequence-specific affinity selection of *EP1* mRNA via *Tb*ZFP3 immunoprecipitation

**Figure 3:**
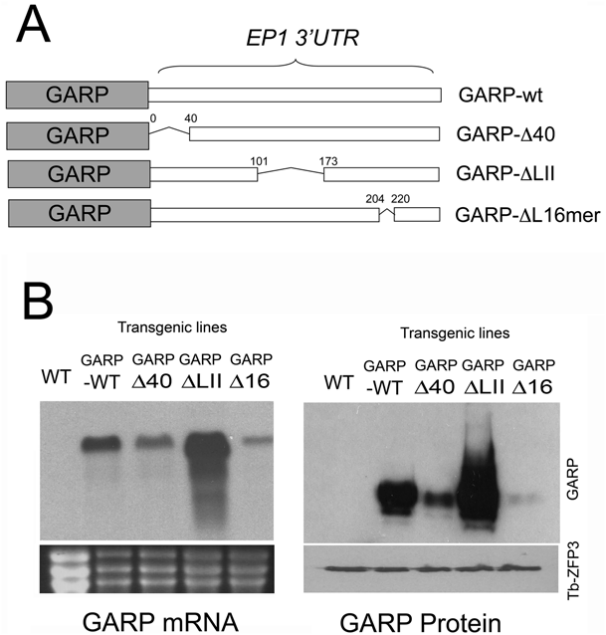
Construction and *in vivo* expression analysis of GARP reporters with mutated *EP1* 3′UTRs. (A) Schematic representation of the reporter constructs used to assay *Tb*ZFP3 selection, highlighting deleted regions in each construct as described in [23]. (B) GARP RNA and protein expression generated in the reporter cell lines transfected with the constructs illustrated in (A). Relative loading is indicated, for Northern blots, by the rRNA levels (revealed by ethidium bromide staining) or, for Western blots, by *Tb*ZFP3 levels.

The sequences which regulate *procyclin* gene expression have been very well characterised in transgenic parasites by use of reporter genes linked to wild type or mutant forms of the *EP1* mRNA 3′ UTR. This has identified a number of regulatory regions that act to either positively or negatively control expression [14], [23]–[26]. Minimally, three domains contribute to *EP procyclin* regulation: a positive control element in the first 40 nt after the stop codon (“Loop I”), a negative element contained within 101–173 nt (‘Loop II’) and a further positive element comprising a highly conserved 16 nt stem loop structure (“Loop III”). To determine whether *Tb*ZFP3 RNA–immunoprecipitation generated sequence-specific selection of *EP1 procyclin* mRNA, we generated a series of cell lines transfected with previously characterised reporter constructs (kindly provided by Professor I. Roditi, University of Bern). These comprised a *GARP* coding region reporter [27] linked to either the wild type *EP1 procyclin* 3′UTR or mutants lacking each regulatory domain (Δ40, ΔLII or Δ16mer) (Figure 3A). Initially the anticipated effects on reporter gene expression for each construct were confirmed by analysing the *GARP* mRNA and protein levels in the resulting transfected cell lines (Figure 3B). Matching previous analyses of these deletions [23], the mRNA abundance of *GARP* was reduced in the Δ40 (62% of wild type levels) and Δ16mer cell lines (26% of wild type levels), but significantly elevated in the ΔLII cell line (210% of wild type levels). Similarly, Western blotting of protein extracts from these cell lines with a GARP antiserum [28] confirmed that the levels of GARP protein translated from the expression constructs matched previous observations, with abundant GARP generated in the ΔLII cell line and little detectable protein when the 16mer element was deleted.

**Figure 4:**
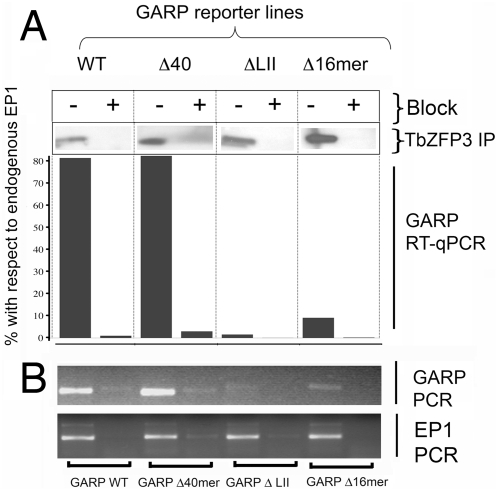
Sequence specific co-precipitation of *EP1* 3′UTR with *Tb*ZFP3. (A) Co-immunoprecipitation of each *GARP* reporter mRNA with *Tb*ZFP3 either in the presence or absence of blocking peptide (“GARP RT-qPCR”). qRT-PCR values are normalised to the value for co-selected endogenous *EP1* mRNA in each cell line. Control amplifications lacking input RNA or reverse transcriptase failed to amplify any product. Above each bar chart is shown Western blots for *Tb*ZFP3 (“*Tb*ZFP3 IP”), using proteins from each immunoprecipitation (plus or minus peptide block) used to isolate *Tb*ZFP3 and bound mRNAs from each cell line. These confirm specificity of the selection. (B) Independent confirmation of the real-time PCR data generated in a separate immunoprecipitation, with the products visualised on ethidium bromide stained agarose gels (“GARP-PCR”). The co-selection of endogenous *EP1* mRNA is also shown (“EP1-PCR”).

Having generated cell lines stably transfected with each reporter construct, extracts from each were subjected to *Tb*ZFP3-immunoprecipitation, either in the presence or absence of blocking peptide and analysed for the selection of *Tb*ZFP3, (Figure 4A, “TbZFP3 IP”) or of the reporter *GARP* mRNA (Figure 4A; “GARP-RT-qPCR”). Importantly, in each case the relative selection of *GARP* mRNA was compared with, and normalised to, the selection of endogenous *EP1 procyclin*, ensuring the efficiency of immunoprecipitation from each extract was equivalent (Figure 4A and 4B). In the cell line containing *GARP* linked to wild type *EP procyclin* 3′UTR, efficient selection of the reporter mRNA was observed with this being abolished in the presence of the blocking peptide (80% and 1% respectively, normalised to the relative immunoprecipitation of endogenous *EP1* mRNA). When the Δ40 cell line was examined efficient selection of *GARP* transcripts was also observed (81% of endogenous *EP1*, with 3% of endogenous *EP1* in the presence of the peptide block). However, when either the negative control element contained in Loop II of the *EP1 procyclin* 3′UTR, or the 16mer stem-loop structure were deleted, selection with *Tb*ZFP3 was reduced to only 1.5% or 9% of endogenous *EP1* mRNA, respectively. This did not represent inefficient immunoprecipitation since endogenous *EP1 procyclin* mRNA was selected at an equivalent level in all cell lines (Figure 4B and data not shown). Moreover, it was not simply dependent on target mRNA abundance because the ΔLII–derived *GARP* mRNA was highly expressed (Figure 3B). This demonstrated that *Tb*ZFP3 immunoprecipitation showed sequence-specific selection of the *EP procyclin* 3′UTR, this being individually dependent upon integrity of the Loop II and the 16mer regulatory domains.

### *Tb*ZFP3 is a positive regulator of EP1 Procyclin operating at the protein level

**Figure 5:**
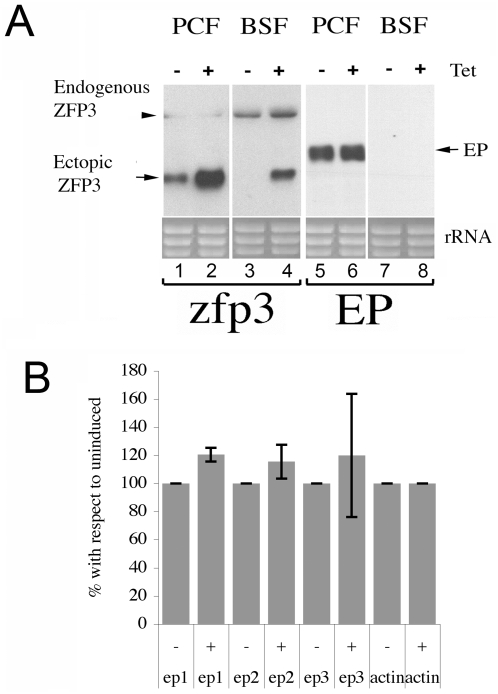
Effect of ectopic over-expression of *Tb*ZFP3 on *procyclin* mRNA levels. (A) Northern blots of procyclic forms (PCF) and bloodstream form (BSF) transgenic trypanosome lines that induce the ectopic expression of *Tb*ZFP3 under a tetracycline regulatable promoter. Lanes 1–4 show the expression of the ectopic *Tb*ZFP3 (arrowed) in the absence or presence of induction. Endogenous *Tb*ZFP3 mRNA is also shown (arrowhead). Lanes 5–8 (“*EP*”) show the same RNAs hybridised with a riboprobe detecting all *EP* procyclin isoforms. The relative loading is indicated by rRNA below each panel. (B) Relative expression of *EP1*, *EP2*, and *EP3* mRNAs after ectopic expression of *Tb*ZFP3 as determined by quantitative RT-PCR. Values are normalised to *actin*. All *procyclin* isoform mRNAs showed an approximately 20% increase in abundance, but no specific isoform showed a consistent change with respect to another. *GPEET mRNA* levels were also not affected (data not shown). Error bars = SD.

**Figure 6:**
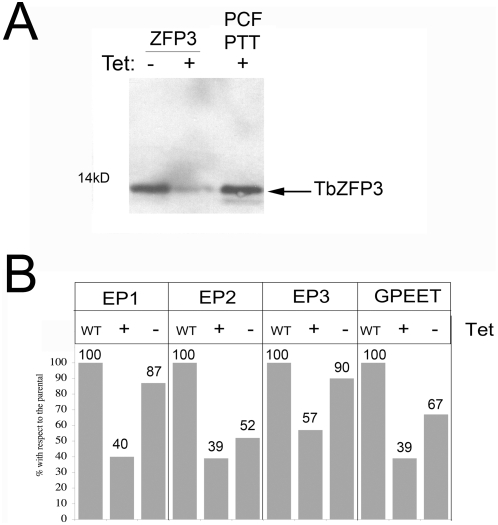
RNAi against *Tb*ZFP3 generates reduced mRNA abundance for all *procyclin* mRNA isoforms. The *Tb*ZFP3 coding region was cloned into the RNAi vector p2T7i [41] and transfected into the RNAi competent procyclic cell line, PTT. Cells were induced to undergo RNAi by growth in medium containing tetracycline (1 µg/ml) for 48 h. Protein extracts from the induced and uninduced samples, and from the parental cell line, were then assayed for *Tb*ZFP3 expression by Western blotting (A). RNAs isolated from the respective samples were then assayed for relative *procyclin* mRNA expression by quantitative RT-PCR (B). All isoforms showed an inducible reduction in abundance in the presence of tetracycline (when TbZFP3 RNAi is induced; “+”). Some reduction was also seen in the absence of tetracycline (“−”) due to leaky RNAi.

Having demonstrated that *EP1 procyclin* mRNA co-selects with *Tb*ZFP3 via known regulatory domains we determined if *Tb*ZFP3 could specifically regulate *EP procyclin* mRNA abundance. Initially, we made use of transgenic procyclic and bloodstream form lines that ectopically over-express *Tb*ZFP3 under tetracycline control. Figure 5A (lanes 1–4) shows endogenous and ectopically expressed *Tb*ZFP3 mRNA in each cell line, whereas lanes 5–8 shows hybridisation to the same RNAs of a generic *EP procyclin* riboprobe. This revealed no evidence for a specific enrichment of any *EP mRNA* in response to *Tb*ZFP3 induction in procyclic forms nor appearance of *EP mRNA* in bloodstream forms (where Procyclin is not normally expressed). Furthermore, quantitative RT-PCR specific for *EP1*, *EP2*, *EP3 procyclin* revealed no specific change of *EP1* mRNA with respect to *EP2* or *EP3* mRNAs, although the expression of all mRNAs increased slightly (∼20%; Figure 5B). Similarly, RNAi directed to *Tb*ZFP3 (resulting in 60% reduction of protein expression; Figure 6A) resulted in no specific regulation of any *procyclin* mRNA isoform, although all *procyclin* mRNAs as well as several housekeeping genes showed an overall reduction of mRNA abundance. This suggests non-specific or indirect effects or, potentially, a more widescale consequence of *Tb*ZFP3 knock-down on mRNA abundance (Figure 6B and data not shown). Nonetheless, the analysis demonstrated that there was no differential change in the abundance of *procyclin* isoform mRNAs caused by enhanced or reduced *Tb*ZFP3 expression.

**Figure 7:**
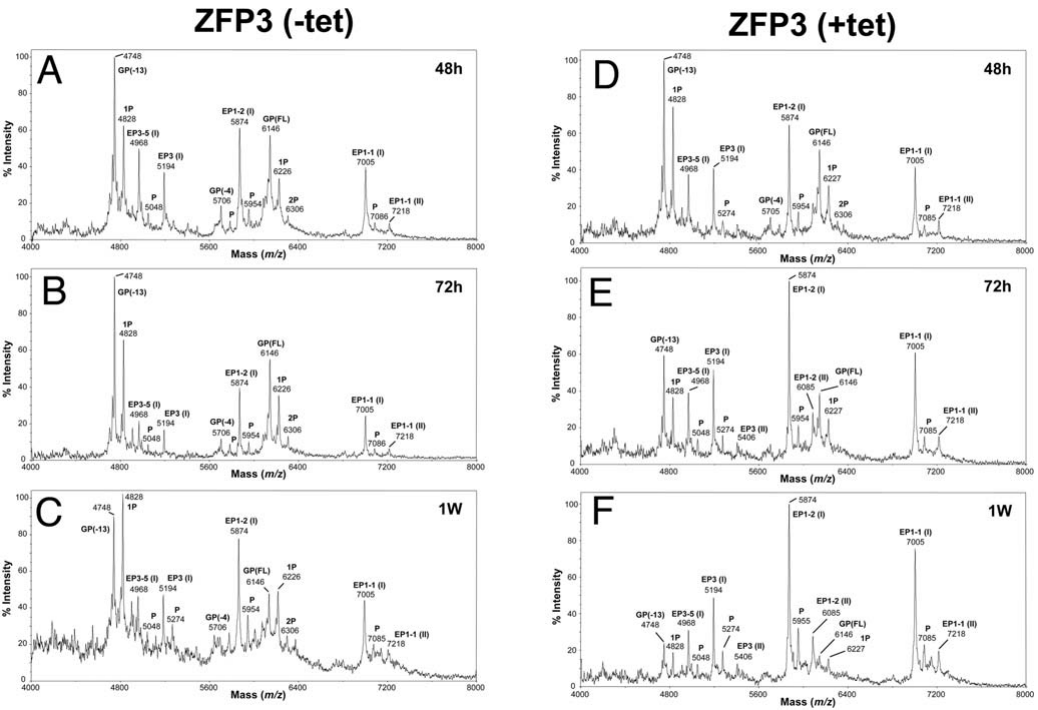
Procyclin isoform regulation by *Tb*ZFP3. Negative ion MALDI-TOF mass spectra of Procyclin isoforms extracted from cells uninduced (A–C) or induced (D–F) to express ectopic *Tb*ZFP3 to a level ∼2-fold its endogenous abundance. In response to elevated *Tb*ZFP3 levels, the expression of EP1-1 and EP1-2 (which share an identical 3′UTR) is elevated, whereas GPEET expression is reduced. (A) and (D) show uninduced and induced cells after 48 h, (B) and (E) show cells after 72 h, and (C) and (F) show cells after 1 week. EP3-5 is an allelic copy of the *EP3* gene in the 427 strain [42]. EP C-terminal polypeptides (I) and (II) represent forms containing the sequence P (EP)_n_G-EtN and PDP (EP)_n_G-EtN, respectively. GP (FL), (-4), and (-13) indicate full-length GPEET and its polypeptides lacking four and thirteen N-termini aminoacids, respectively. P, indicates levels of peptide phosphorylation.

To monitor the relative protein expression of individual Procyclin isoforms we made use of an established mass spectrometry approach to detect Procyclins. Thus, cell lines induced to ectopically express *Tb*ZFP3 for 48 h, 72 h or 1 week were subject to delipidation and butanol extraction followed by aqueous HF treatment to release the Procyclin proteins from their GPI-anchors. The released full length Procyclins were then further subject to mild acid treatment [29], a procedure that cleaves the EP isoforms at the Asp-Pro bonds and partially cleaves GPEET between Asp-Gly. The resulting extracts were analysed by negative ion MALDI-TOF-MS to detect the presence and abundance (based on their comparable ionisations) of the [M-H]^−^ pseudomolecular ions representing C-terminal fragments of GPEET and EP1-1, EP1-2, EP2 and EP3 proteins. Consistent with expectation for this parasite strain, the dominant surface protein was GPEET Procyclin [20],[21], with lower expression of the EP Procyclin isoforms (Figure 7A–7C). However, ectopic expression of *Tb*ZFP3 (generating 1.5 and 2.3 fold overexpression at 48 h and 72 h, respectively), progressively elevated EP1-1 and EP1-2 Procyclins (Figure 7D–7F) above either uninduced controls (Figure 7A–7C) or the parental cell line grown in the presence of tetracycline (Figure S1). Furthermore, expression of GPEET was progressively reduced (∼5 fold) from being the dominant surface molecule to being a minor component with respect to EP1 after 7 days of induction (Figure 7F). In contrast to these two proteins, both allelic variants of EP3 procyclin (EP3-1 and EP3-5) remained relatively unchanged, whereas EP2 was not detected in any cell population matching previous studies. Consistent with the specific regulation of Procyclin expression by *Tb*ZFP3, examination of the Procyclin protein signature of the cell line expressing the ΔCCCH mutant of *Tb*ZFP3, which does not co-select *procyclin* mRNAs, did not reveal any change in the profile of expressed proteins regardless of whether the ectopic expression of the mutant protein was induced or not (Figure S2).

**Figure 8:**
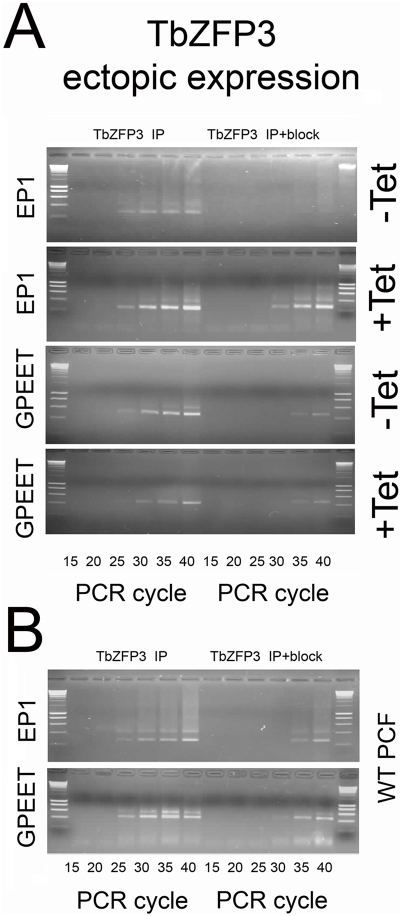
Ectopic expression of *Tb*ZFP3 alters its relative association with *EP1* and *GPEET* mRNAs. (A) RNA-immunoprecipitation of *EP1* and *GPEET* mRNAs by *Tb*ZFP3 specific antibody (in the presence or absence of blocking peptide). Immunoprecipitations were carried out using extracts from cells uniniduced (−Tet) or induced (+Tet) to ectopically express *Tb*ZFP3. Semi-quantitative assays were performed to directly visualise the amplified products, with samples being isolated after 15, 20, 25, 30, 35, and 40 cycles. The efficiency of *EP1* cDNA amplification is enhanced upon *Tb*ZFP3 ectopic expression, whereas the efficiency of *GPEET* cDNA amplification is reduced. All samples were amplified in parallel, resolved on matched gels, and captured at identical exposures. The data are representative of three independent assays. (B) The equivalent assays were also carried out on wild-type procyclic forms as controls. For each reaction, samples generated in the absence of reverse transcriptase failed to amplify any product for *EP1* or *GPEET*, thereby confirming the absence of contaminating genomic DNA (data not shown).

To examine the basis of the altered expression of EP1 and GPEET Procyclins after *Tb*ZFP3 ectopic expression, we assayed the relative co-immunoprecipitation with *Tb*ZFP3 of each *procyclin* mRNA isoform in the *Tb*ZFP3-uninduced population or after 72 h induction. Figure 8 shows a representative semi-quantitative analysis of the relative selection of *EP1* and *GPEET* mRNA in each cell population. This reveals that the relative selection of *EP1* mRNA increased upon *Tb*ZFP3 ectopic expression, whereas the efficiency of *GPEET* mRNA selection was diminished. We conclude that moderate elevation of *Tb*ZFP3 levels alters the relative association with *EP1* and *GPEET* mRNAs, this resulting in a change of trypanosome surface antigen expression, inducing a change from GPEET to EP1 Procyclin as the dominant surface protein on procyclic forms.

## Discussion

The experiments in this paper identify the developmental regulator, *Tb*ZFP3, as an isoform-specific regulator of Procyclin surface coat expression in trypanosomes. Specifically, we demonstrate (i) that endogenous *Tb*ZFP3 shows sequence-specific co-association with distinct *procyclin* mRNA isoforms, (ii) that ectopic overexpression of *Tb*ZFP3 does not enhance the mRNA abundance of selected transcripts, but rather that (iii) their protein expression is regulated in an isoform-specific manner, as evidenced by mass spectrometric analysis of the Procyclin expression signature in transgenic cell lines. Unlike the wild type *Tb*ZFP3 protein, a mutant form of *Tb*ZFP3 lacking its C×8C×5C×3H predicted RNA-interaction motif and which cannot co-associate with *procyclin* mRNAs does not alter Procyclin expression. We have already demonstrated that *Tb*ZFP3 promotes differentiation when associated with the translational machinery (this being dependent upon its predicted RNA and protein interaction motifs) and that this occurs only in the parasite life cycle stage at which Procyclin proteins are expressed [18]. Hence, our work provides a long-sought ‘missing link’ between the intensely studied *cis*-regulatory signals for the *procyclin* gene family and the general gene expression machinery, this being the translational apparatus.

*Tb*ZFP3 shows specific co-association *in vivo* with *EP1* and *GPEET procyclin* mRNA, whereas the distinctly regulated transcripts *EP2* and *EP3* are not co-immunoprecipitated. Although deletion of the predicted RNA-binding domain in *Tb*ZFP3 prevents the co-association with *EP1* mRNA, our studies do not formally distinguish between direct intermolecular contact between *Tb*ZFP3 and target mRNAs and indirect contact dependent on other protein factors. Hence, we use the term *Tb*ZFP3mRNP to define the composition of the immunoprecipitated material comprising *Tb*ZFP3, *procyclin* mRNAs and, possibly, other identified (*e.g.* [18]; see below) and unidentified co-operating factors. Nonetheless, by using an immunoprecipitation approach employing an antibody directed to the endogenous *Tb*ZFP3 protein in wild type parasites we demonstrate that the observed co-association with *procyclin* mRNAs is physiological, and directed by *Tb*ZFP3 in its normal cellular context. Interestingly, the differential selection of different *procyclin* mRNA isoforms by the *Tb*ZFP3mRNP matches their overall sequence similarity, with *EP1* and *GPEET* 3′UTR sequences being significantly more closely related than *EP2* and *EP3* (Figure S3). Nonetheless, EP1 and GPEET are differentially regulated *in vivo*, with GPEET expression being repressed as EP1 is upregulated during differentiation to late procyclic forms *in vitro* and in the tsetse fly [8],[12]. Significantly, this matches the observed effects of *Tb*ZFP3 ectopic overexpression, whereby EP1 expression is elevated to become the dominant surface protein and GPEET expression is repressed, this correlating with enhanced association of the *Tb*ZFP3mRNP with *EP1* mRNA and diminished association with *GPEET* mRNA. Although copy number control of Procyclins on the parasite surface could accentuate this switch, it is significant that the inverse control of these surface molecules is regulated by only subtle changes in the abundance of *Tb*ZFP3 (∼1.5–2.5 fold). This suggests exquisitely regulated control of Procyclin isoform expression in response to *Tb*ZFP3 levels.

**Figure 9:**
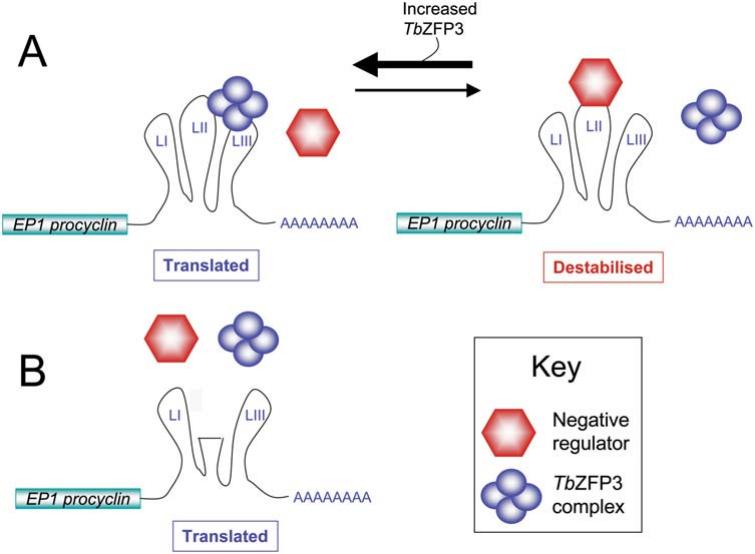
Model for the function of *Tb*ZFP3 in EP1 Procyclin expression. (A) The expression of *EP1 procyclin* is governed by the balance between a negative regulator (hexagons) and a *Tb*ZFP3-containing mRNP complex (circles), each of which competes for binding at the Loop II region. When bound by the negative regulator, *EP1 procyclin* is poorly translated and its mRNA destabilised. When *Tb*ZFP3 expression is increased by ectopic over-expression, the negative regulator is out-competed and expression of EP1 Procyclin increases. (B) When the Loop II region is deleted, neither *Tb*ZFP3 nor the negative regulator can bind. In the absence of either factor, EP1 Procyclin expression is enhanced above wild type levels. This demonstrates that *Tb*ZFP3 binding is not required for efficient expression, instead suggesting that the *Tb*ZFP3 complex acts as an anti-repressor.

In addition to isoform-specific selection, the *Tb*ZFP3mRNP exhibits sequence-specific association with the *EP1* mRNA 3′UTR, this being dependent upon the integrity of two well-characterised regulatory regions - the ‘Loop II’ and the 16mer stem loop region. Previous analyses have demonstrated that these sequences provide negative and positive control elements for *EP1 procyclin* expression, respectively. The Loop II region acts as a translational repressor and mRNA destabilisation element in procyclic forms, whereas the 16mer is a translational enhancer, which suppresses the action of the Loop II region [23],[24]. In insect stages, it was predicted that a macromolecular complex would associate with both elements and so shield the ‘Loop II’ element from recognition by a negative regulator, thereby promoting gene expression [23]. Our findings are compatible with this, invoking a model (Figure 9) in which *Tb*ZFP3 competes with a negative regulator binding ‘Loop II’, such that *Tb*ZFP3 over-expression promotes EP1 Procyclin expression (at the expense of GPEET), whereas RNAi mediated removal of *Tb*ZFP3 results in reduced *procyclin* mRNA abundance. Interestingly, expression of the Loop II deletion reporter construct revealed that *Tb*ZFP3mRNP-binding is not necessary for efficient mRNA or protein expression (Figure 3B), suggesting that the *Tb*ZFP3mRNP acts primarily as an anti-repressor (Figure 9), matching earlier predictions for the *procyclin* regulatory machinery [2],[17],[23],[30]. This is analogous to the regulation of *nanos* RNA during *Drosophila* embryogenesis, whereby overexpression of Oskar displaces the translational repressor Smaug bound to the *nanos* 3′UTR [31].

CCCH proteins in eukaryotes are involved in all levels of gene expression through RNA recognition, usually this being dependent upon the integrity of at least two juxtaposed CCCH fingers [32]. *Tb*ZFP3, however, has only a single CCCH motif and is a member of a family of small CCCH zinc finger proteins (*Tb*ZFP1, *Tb*ZFP2 and *Tb*ZFP3), unique to kinetoplastids, and each <140 amino acids long. These proteins interact in yeast 2-hybrid assays and co-immunoprecipitate *in vivo*, suggesting that they provide a modular function in order to confer specificity of binding [33]. Whether this is part of a single mRNP complex, or distinct complexes with differential specificities for different genes or in different life cycle stages, remains to be determined. This complexity of interactions may define the specificity of different mRNA classes selected by the *Tb*ZFP3mRNP, or moderate their differential efficiency of selection and hence regulation, as observed with *EP1* and *GPEET* mRNA regulation under conditions of *Tb*ZFP3 ectopic expression. To be understood in depth, such interactions will need to be analysed on a case-by-case basis for individual RNAs as has been done here for *procyclin* mRNAs. Nonetheless, analogous combinatorial interaction between RNA binding proteins in kinetoplastid parasites to confer target specificity and regulation has previously been proposed for the small RNA binding proteins *Tb*UBP1 and *Tb*UBP2 [34], homologues of the regulators of *mucin* gene expression in *T.cruzi* [35].

*Tb*ZFP2 and *Tb*ZFP3 are constitutively expressed and associate with the translation apparatus in procyclic forms but not in bloodstream forms [18]. Our demonstration here that the *Tb*ZFP3mRNP co-associates with *procyclin* mRNA regulatory elements that control translation, thus promoting EP1 surface protein expression without enhancing *EP1* mRNA abundance, suggests a role for *Tb*ZFP3 in translational control. Supporting this, a mutant *Tb*ZFP3 lacking the predicted RNA-interaction domain neither co-associates with *procyclin* mRNAs (Figure 2) nor the translational apparatus [18] and induces no consistent change in Procyclin expression (Figure S2). Temporally-regulated translational control is a key aspect of cell-type development in the *Plasmodium* parasite, whereby translational repression *via* the DDX6 RNA helicase family member DOZI regulates gametocyte mRNA expression and life-cycle differentiation [36]. Interestingly, these transcripts share a 47 nt U-rich control element [37], similar to the regulatory U-rich 26mer elements enriched in procyclic form-specific transcripts [38] and comprising part of the Loop II region of *EP1 procyclin* recognised by *Tb*ZFP3. This points to common mechanisms of developmental control among widely divergent eukaryotic protozoan pathogens.

Translational control is believed to be a major mechanism of gene regulation in trypanososmatid parasites [39],[40]. Although the general mRNA degradation and translational machineries are broadly conserved in these evolutionarily ancient eukaryotic organisms [3], it is the kinetoplastid-specific *trans*-acting regulators that provide the key to understanding their extreme emphasis on post-transcriptional control. Moreover, targeting unique components of the translational machinery in pathogens is a major strategy in antimicrobial therapies. Thus, discovering novel regulators interacting with this apparatus provides both new understanding of gene expression and new possibilities to intervene in the virulence and spread of these devastating parasites.

## Materials and Methods

### Cell Lines

Procyclic form or bloodstream form *T. brucei* Lister 427 trypanosomes were used throughout. Cell lines engineered for *Tb*ZFP3 ectopic expression in procyclic or bloodstream forms have been described previously and were cultured in SDM-79 or HMI-9, respectively [18].

### Immunoprecipitation and qRT-PCR

Immunoprecipitation using *Tb*ZFP3-specific antisera, RNA extraction and reverse transcription have been described previously [18]. Blocking peptides used were N-DSSQMQQVGHDVPPMA-C for *Tb*ZFP3 and N-EVHTNQDPLD-C for Ty1, each being titrated prior to use. SYBR green qRTPCR reactions were performed using Roche reagents as per specifications for the LightCycler system. The 5′ primers for *actin*, *ep* and *gpeet* and 3′ primers specific to *EP1*, *EP2*, *EP3*, *GPEET*, or the Anchor sequence were described previously [11],[18]. cDNA was amplified as follows: 10 min, 95°C; 30×[8 s, 95°C; 9 s, 55°C; 12 s, 72°C] with fluorescence acquired at 82°C. The amplification was followed by a melting temperature analysis that measured PCR product fluorescence during a temperature increase from 65°C to 95°C at 0.1°C/s to determine product melting temperature and confirm specificity. Product identities were further verified by gel electrophoresis and DNA sequencing. In all cases, serial dilutions of input cDNAs confirmed the quantitative efficiency of the reactions and “no reverse transcriptase” controls confirmed the absence of contaminating genomic DNA in the RNA preparations.

### Northern and Western blotting

Northern blotting involved resolution of 3–5 µg of total trypanosome mRNA on formaldehyde agarose gels resolved in MOPS buffer. Hybridization of blots used digoxigenin labelled riboprobes, detected using anti-DIG alkaline phosphatase-conjugated antibody and visualised using CDP-star as a reaction substrate (Roche). Western blots were detected and quantitated using a Li-COR Odyssey system, using alpha-tubulin as an internal standard.

### Mass spectrometry

Mass spectrometry was carried out according to the methodology described in [8],[19],[29]. Briefly, parasite pellets were freeze-dried and then extracted twice with 200 µl of Chloroform/Methanol/Water, 10∶10∶3 (V/V/V), under sonication (10 min). After centrifugation, the delipidated pellets were then extracted 3 times with 150 µl of 9% butanol (ButOH), also under sonication. The ButOH fractions contain the Procyclins. All ButOH fractions were then freeze-dried and submitted to dephosphorylation using 50 µl of 48% aqueous hydrofluoric acid (aq.HF), at 0°C for 24 h. After aq.HF incubation the samples were freeze-dried again and washed twice with water. The samples were then dried and further incubated with 200 µl of 40 mM TFA, 20 min at 100°C (mild acid conditions), in order to assist visualization of the Procyclin C-termini and the identification of each isoform. Under this condition, the Asp-Pro bonds of most of the EP isoforms are cleaved whereas GPEET partially releases 13 amino acids at its N-terminus. Equivalent amounts of each sample were mixed with α-cyano (matrix) and analysed by negative-ion MALDI-TOF-MS using a Voyager-DE STR instrument.

## Supporting Information

Figure S1 Procyclin isoform expression in parental procyclic forms.
(0.51 MB DOC)

Figure S2 Procyclin isoform expression in transgenic cells expressing the ΔCCCH mutant of *Tb*ZFP3-Ty.
(0.58 MB DOC)

Figure S3 Overall similarity between the 3′UTRs of *EP1*, *EP2*, *EP3*, and *GPEET procyclin* mRNAs.
(2.22 MB DOC)
