# Supplementary material for: Differential Trypanosome Surface Coat Regulation by a CCCH Protein That Co-Associates with procyclin mRNA cis-Elements
Source: PLoS Pathog. 2009 Feb 27;5(2):e1000317. doi: 10.1371/journal.ppat.1000317 (PMC2642730; doi:10.1371/journal.ppat.1000317)
Supplement: Figure S1 — Procyclin isoform expression in parental procyclic forms. (0.51 MB DOC) [file ppat.1000317.s001.doc]

**Supplementary information for Walrad, Paterou, Acosta-Serrano and Matthews.**

**Supplementary Figure 1.**

*Procyclin isoform expression in parental procyclic forms.*

Negative ion MALDI-TOF mass spectra of Procyclin isoforms extracted from Parental 449 in the presence (+tet) or absence (-tet) of 1µg/ml tetracycline. No significant changes in the type and abundance of Procyclins after addition of tetracycline or during the time course of growth were observed. As presented in Figure 7, EP3-5 is an allelic copy of the *EP3* gene in the 427 strain (Hall et al., 2005). EP C-terminal polypeptides **(I)** and **(II)** represent forms containing the sequence P(EP)_n_G-EtN and PDP(EP)_n_G-EtN, respectively. **GP (FL)**, **(-4)** and **(-13)** indicate full length GPEET and its polypeptides lacking four and thirteen N-termini amino acids, respectively. **P**, indicates levels of peptide phosphorylation.

**Parental 449 Procyclic forms**

**
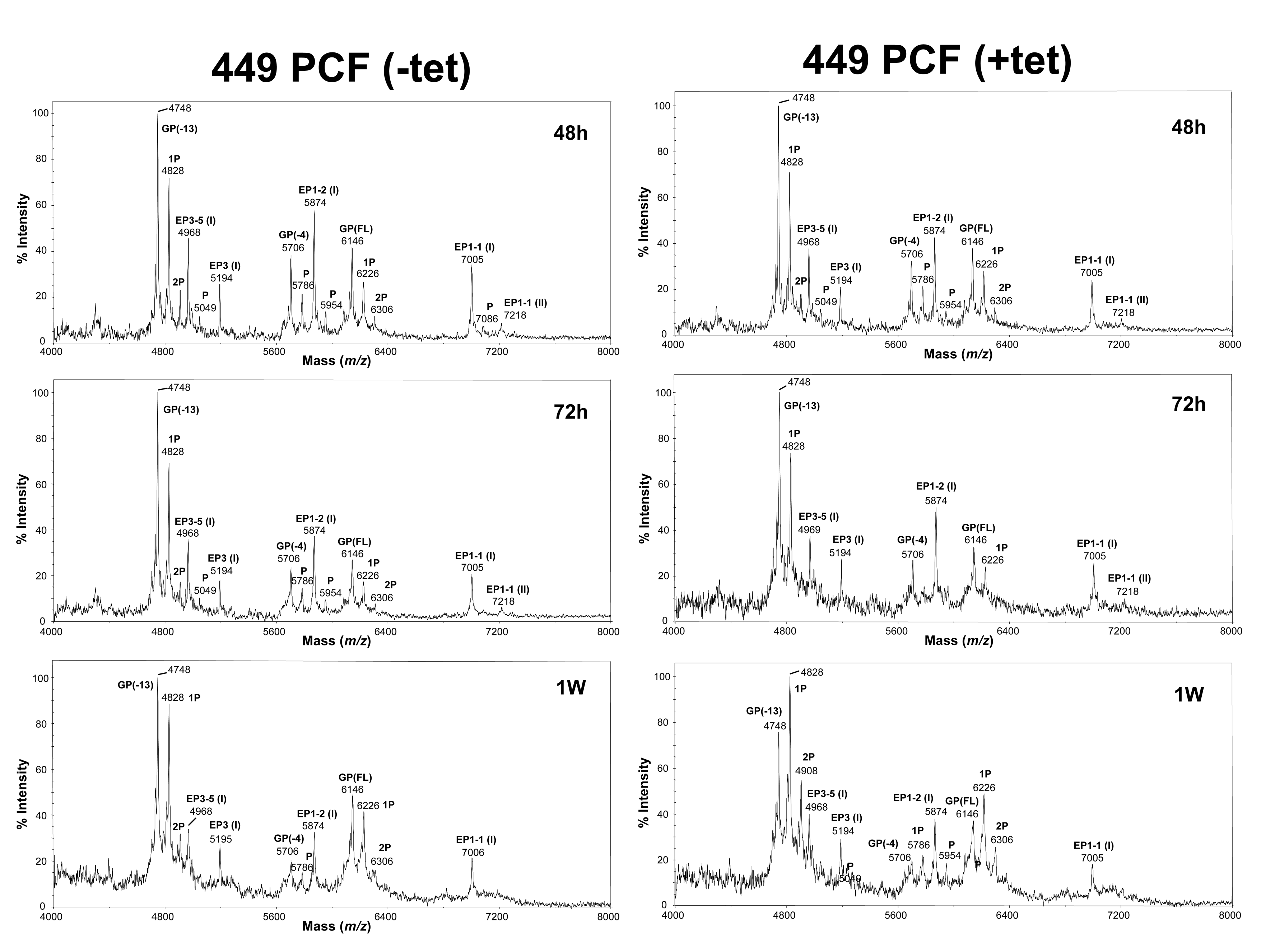
**
