# Supplementary material for: Differential Trypanosome Surface Coat Regulation by a CCCH Protein That Co-Associates with procyclin mRNA cis-Elements
Source: PLoS Pathog. 2009 Feb 27;5(2):e1000317. doi: 10.1371/journal.ppat.1000317 (PMC2642730; doi:10.1371/journal.ppat.1000317)

**Supplementary information for Walrad, Paterou, Acosta-Serrano and Matthews.**

**Supplementary Figure 3.**

Overall similarity between the 3’UTRs of *EP1, EP2, EP3* and *GPEET* *procyclin* mRNAs. The sequences of each 3’UTR are also shown, with the regions deleted in the various *EP1* 3’UTR reporter constructs boxed.

**
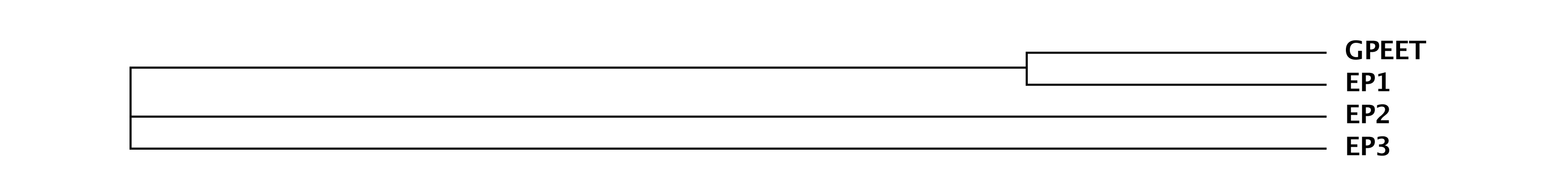
**


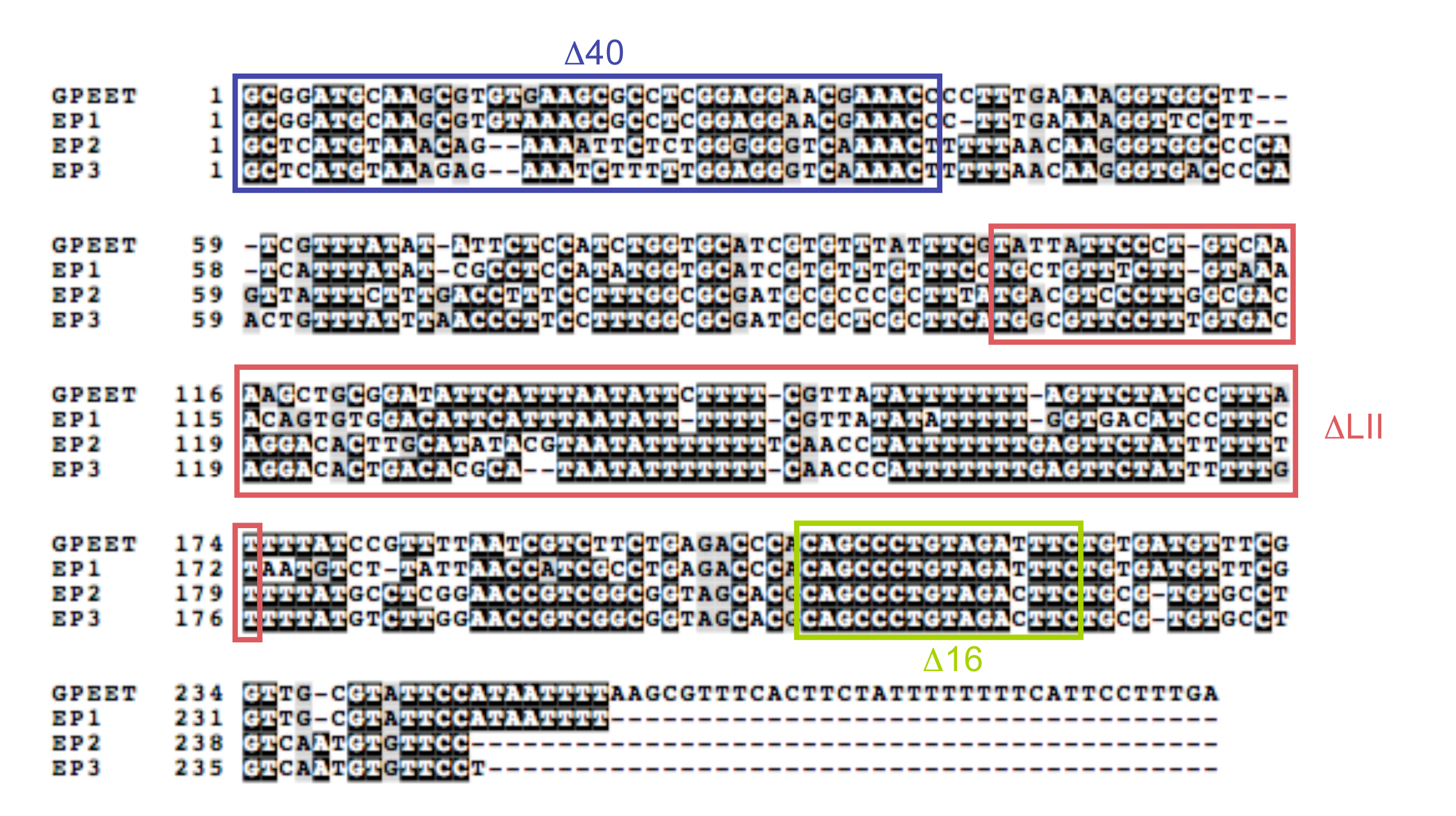

Supplement: Figure S3 — Overall similarity between the 3′UTRs of EP1, EP2, EP3, and GPEET procyclin mRNAs. (2.22 MB DOC) [file ppat.1000317.s003.doc]
